# Bidirectional Associations between Restrained Eating and Body Mass Index in Middle Childhood

**DOI:** 10.3390/nu13051485

**Published:** 2021-04-28

**Authors:** Meg Lawless, Lenka H. Shriver, Laura Hubbs-Tait, Glade L. Topham, Taren Swindle, Amanda W. Harrist

**Affiliations:** 1 Department of Nutrition, University of North Carolina Greensboro, 319 College Avenue, Greensboro, NC 27412, USA; m_lawles@uncg.edu; 2 Department of Human Development & Family Science, Oklahoma State University, 341 Human Sciences, Stillwater, OK 74078, USA; laura.hubbs@okstate.edu; 3 Department of Applied Human Sciences, Kansas State University, 101 Campus Creek Complex, Manhattan, KS 66506, USA; gtopham@ksu.edu; 4 Department of Family and Preventive Medicine, University of Arkansas for Medical Science, 4301 West Markham, Little Rock, AR 72205, USA; TSwindle@uams.edu; 5 Department of Human Development & Family Science, Oklahoma State University, 244 Human Sciences, Stillwater, OK 74078, USA; amanda.harrist@okstate.edu

**Keywords:** dietary restraint, body mass index, middle childhood, childhood obesity

## Abstract

The nature of the association between dietary restraint and weight has been examined in adult samples, but much less is known about this relationship among children. The current study examined the transactional associations among restrained eating behavior and weight among boys and girls during middle childhood. Data for this study came from 263 children participating in the Families and Schools for Health Project (FiSH), a longitudinal study of the psychosocial correlates of childhood obesity. Participants were interviewed by trained researchers in their third- and fourth-grade year when they completed questionnaires and anthropometric assessments. Dietary restraint was assessed using the restrained eating subscale of the Dutch Eating Behavior Questionnaire (DEBQ), and weight was assessed using Body Mass Index z-scores (BMIz). Bidirectional associations between variables were examined using cross-lagged models controlling for children’s sex, ethnicity, and weight in first grade. Results indicated that weight in grade 3 was related to greater dietary restraint in grade 4 (B = 0.20, *p* = 0.001), but dietary restraint in grade 3 was not associated with weight in grade 4 (B = 0.01, *p* = 0.64). Neither child sex nor race/ethnicity were associated with BMIz or dietary restraint at either time point. Findings from this study advance the existing limited understanding of eating behavior development among children and show that weight predicts increases in children’s dietary restraint in middle childhood.

## 1. Introduction

Recent projections estimate that over 50% of adults will be obese by 2030, a staggering number considering the health and financial burden associated with excess adiposity [1,2]. Further, children who are overweight and obese are more likely to remain overweight as adults [3]. Strategies for reversing obesity and associated comorbidities in adulthood have, so far, been limited in their long-term success [4,5,6]. Thus, prevention of overweight and obesity is integral to current public health efforts to reduce the burden of weight-related diseases on society [7]. Identification of effective childhood weight management strategies is needed to improve obesity prevention efforts.

Dietary restraint, or the conscious control of food intake for the purpose of weight management, has been a controversial topic in the literature with regard to its association with weight gain and other negative nutrition outcomes [8,9,10,11]. On one hand, there is evidence that excessive dietary restriction may alter metabolic functioning, making it difficult for individuals to maintain long-term weight loss [12]. This notion is supported by findings from some longitudinal studies, which indicate that adolescents and adults who reported dieting and weight loss efforts at baseline showed greater weight gain at follow-up and an elevated risk of obesity [13,14]. The potential mechanism for these findings has been attributed to the fact that individuals who report dieting and weight loss attempts engage unsustainable forms of dietary restraint that leads to binge eating episodes and weight gain over time [15]. Experimental investigations, however, have consistently shown that dietary restrainers are not in negative energy balance, which suggests that dietary restraint is not an accurate indicator of actual restriction of food intake [11,16].

Another line of research has found that increasing dietary restraint can be effective in successfully preventing weight gain in adults, particularly among individuals prone to overeating [4,17]. This ability might be especially important in the current food environment, which is characterized by a wide availability of highly palatable, energy dense foods over healthier and more nutrient-dense options [18]. Moreover, there is evidence that difficulties with self-control of energy intake is associated with poor dietary intake and weight status in children [19,20,21]. Thus, it is possible that some degree of dietary restraint is important as an adaptive measure to prevent and/or minimize weight gain over time [11,22].

To date, dietary restraint in children has been studied for its association with early maternal feeding practices, child self-esteem, and child body dissatisfaction [23,24,25,26,27]. Limited research has focused on prospective associations between dietary restraint and weight status in children [28]. The most common measure used for assessing dietary restraint in these studies has been the restraint subscale of the Dutch Eating Behavior Questionnaire [29,30]. Previous research has indicated its use for children as young as 7 years as this is the age when measures of dietary restraint begin to show acceptable levels of reliability and validity [31]. Because weight-related concerns and dieting behaviors have been observed in studies with children as young as 5 years old, additional evidence for the nature of the relation between dietary restraint and weight in middle childhood is currently warranted [32,33].

Bidirectional influences of weight and restraint have been conceptualized in the literature on eating behaviors; however, empirical research examining these dynamic relations in child and adolescent samples remains limited [34,35,36,37]. To our knowledge, no study has investigated the direction of the relation between dietary restraint and weight during middle childhood, a period that has been identified as a key developmental period for the onset of eating disorder symptomology and subsequent weight gain [28,33,38]. The current study builds on previous prospective research by examining the transactional associations among restrained eating behavior and weight among school-aged boys and girls. Specifically, we consider the longitudinal indirect pathways by which restrained eating behavior and weight influence one another in middle childhood. Given the established cross-sectional link between early weight status and emerging dietary restraint, it is hypothesized that greater weight in grade 3 will predict higher levels of dietary restraint in grade 4, whereas levels of dietary restraint in grade 3 will be unrelated to weight one year later.

## 2. Materials and Methods

### 2.1. Study Design and Procedures

Data for this study come from participants enrolled in a large-scale longitudinal study examining the social and emotional correlates of childhood obesity titled the Families and Schools for Health (FiSH) project [39,40]. A complete description of the study design has been described elsewhere [39,40,41,42]. Briefly, a community sample of nearly 1200 children and their parents were recruited from 29 elementary schools in rural Oklahoma beginning when children were entering the first grade, and children were followed through the end of fourth grade [39]. Initial written consent to participate in the study was obtained from school principals, teachers, and parents prior to the start of data collection. Children were asked for assent to participate in data collection. Participation in the FiSH project averaged 55.6% per first-grade class (SD = 18.7%) [40,42]. The average proportion of children on free or reduced-price lunch—a proxy for poverty at the school level—was 65% [40]. The study protocol was reviewed and approved by the University Institutional Review Board prior to any data collection.

### 2.2. Procedures

Data from children at the participating schools was collected via individual child interviews and assessments conducted by trained researchers [42]. The interviews took place during regular school days and regular class times [42]. Additionally, all participating parents were mailed a survey and were asked to complete and return questionnaires via mail (only sociodemographic data provided by parents are included in the current study) [40]. If parents did not respond to the child ethnicity question or did not return questionnaires, a research assistant traveled to each school with a form to obtain the child ethnicity data, bringing information on parent provision of consent for demographic data, and obtained the ethnicity data from the school office. A total of 58% of parents did not complete the parent questionnaire packet in the beginning of the study. For these children, information on child sex was recorded by research assistants during the one-on-one child interviews at school [42]. The current study utilizes the following data that were collected during the FiSH study: (1) sociodemographic data (collection method: parent surveys administered at the beginning of 1st grade for all participating children); (2) child BMI (collection method: anthropometric assessment of height and weight in grades 1, 3, and 4); and (3) dietary restraint (collection method: self-report measure administered to children by trained researchers during individual interviews in grades 3 and 4).

### 2.3. Study Measures

#### 2.3.1. Dietary Restraint

Dietary restraint in the current study was assessed using the Dutch Eating Behavior Questionnaire (DEBQ; English version) [29]. This measure contains 33 items, with three factors: restrained, emotional, and external eating. The restrained eating subscale contains 10 items and measures behavioral strategies to control energy intake. Dietary restraint was assessed in a subsample of participants beginning when children were in grade 3 (approximate age 8). Some modifications were made to the original measure to make it more suitable for younger children as demonstrated by previous research in elementary school children [31]. For example, the response set was reduced from a 5-point scale (ranging from seldom to very often) to a 3-point scale (yes, sometimes, no) and the language was simplified (ex. ‘Do you deliberately eat less in order not to become heavier?’ was changed to read ‘Do you try to only eat a little bit on purpose so that you won’t get fat?’) [31]. The Cronbach’s alphas were 0.82, 0.85 in grades 3 and 4, respectively. Only participants with complete data in grades 3 and 4 were included in this study.

#### 2.3.2. Weight and Weight Status

Children’s weight and height were assessed during the interview visits at the participating schools. Trained researchers completed the assessments using standard anthropometric measurement procedures [43]. Weight was measured using a portable digital scale (Shorr Productions, Olney, MD, USA) to the nearest 0.2 pounds. Height measurements were taken twice using a portable height board and recorded to the nearest 0.2 cm; if values were not within ±0.3 cm, a third measure was taken. The average of the recorded values was used to calculate final height and weight for each child. Weight was converted into kilograms and divided by the height in meters squared to calculate body mass index (BMI). BMI-for-age percentiles (BMIp) and z-scores (BMIz) were calculated by expressing a child’s BMI relative to children of the same sex and age in the CDC growth charts [44,45]. The CDC growth references have been designed to reflect growth of children in the U.S. and are recommended for use in children aged 2–20 years of age. For descriptive purposes, children’s weight status was classified utilizing the BMIp and established cut offs as follows: obese (BMI-for-age percentile ≥ 95), overweight (BMI-for-age percentile ≥ 85 and <95) and not overweight or obese (BMI-for-age percentile < 85) [44,45]. BMIz was used to assess child’s weight change over time in the bidirectional models. The advantages of utilizing BMIz as a continuous variable of child growth for population-based assessment have been described elsewhere [46,47,48].

### 2.4. Statistical Analysis

Preliminary analyses were conducted to examine descriptive variables and correlations among the study variables using IBM SPSS Statistics for Windows (Version 26.0). Associations between dietary restraint and BMIz were estimated using Mplus [49]. Full information maximum likelihood (FIML) estimation was used to handle incomplete data. FIML estimation uses all available information to account for missing data. Given the well-established associations between dietary restraint and weight with race, sex, and early weight status, children’s race/ethnicity, sex, and weight in first grade were examined as covariates in all models [25,50,51,52].

Following procedures developed by De Jonge et al. (2001), a stability model without cross-lagged structural paths (model 1) was compared to the more complex, cross-lagged models with structural paths from BMIz and dietary restraint scores in Grade 3 to dietary restraint scores and BMIz, Grade 4, respectively [53]. Model 2 extended model 1 by adding cross-lagged paths from Grade 3 dietary restraint to Grade 4 BMIz and model 3 considered the reverse relationships of Grade 3 BMIz to Grade 4 dietary restraint. Finally, model 4 included all autoregressive and cross-lagged paths from model 1–3. Across models, concurrent associations among constructs were estimated. The model fits were evaluated using several fit indices. A Root Mean Square Error of Approximation (RMSEA) of 0.06 or smaller, a comparative Fit Index (CFI) of 0.95 or larger, a Standardized Root Mean Square Residual (SRMR) of 0.08 or smaller indicate adequate model fit [54,55]. The difference between the nested models were compared by a chi-square difference test.

## 3. Results

A total of 266 children provided data at both time points (grade 3 and 4). Three outliers with potential measurement errors for height and/or weight were removed prior to the final analyses. Baseline characteristics of the study cohort are presented in Table 1. In the final dataset with complete data from grade 3 and 4 (*n* = 263), 56.7% were male, and 18.1% were American Indian with the rest primarily of European American descent. When children were in the third grade, around 62% were normal weight; 17% were overweight; and 21% were in the obese weight status category. A small subsample of children (*n* = 16) in the current study participated in an intervention that was part of a larger study when they were in the first grade. The intervention included components addressing family dynamics, emotion, and behavior regulation, family lifestyle behaviors, and peer acceptance related to child obesity. However, preliminary analyses revealed no significant differences in model results between these children and the rest of the sample.

The means and standard deviations for children’s dietary restraint and BMIz at the two time points are presented in Table 2. Sample means for BMIz and dietary restraint showed stability from the 3rd grade to 4th grade. Significant positive associations were found between BMIz and dietary restraint at both time points (Table 3). Neither child sex nor race/ethnicity were associated with BMIz or dietary restraint at either time point.

### 3.1. Structural Model Comparisons

#### 3.1.1. Stability Model

**Figure 1:**
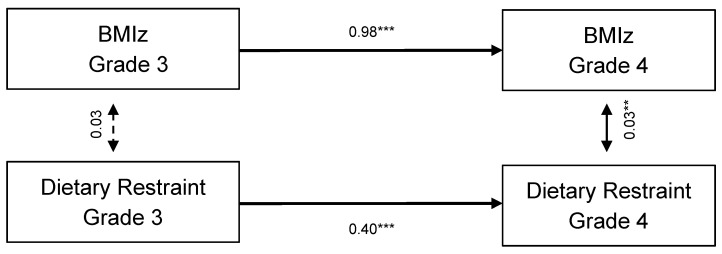
Stability model with BMIz and dietary restraint between grades 3 and 4. ** *p* < 0.01; *** *p* < 0.001.

The stability model (model 1) had good fit to the data, χ^2^(8)= 15.84, *p* = 0.04, comparative fit index (CFI) = 0.99, root mean square error of approximation (RMSEA) = 0.06, and a standardized root mean square residual = 0.06 (see Table 4). Autoregressive coefficients were constant over time for the stability model estimating dietary restraint and weight (see Figure 1). The standardized path coefficients for dietary restraint demonstrated moderate stability and were significant (B = 0.40 *p* < 0.001), indicating scores for dietary restraint were moderately stable between grade 3 and grade 4. For weight, the autoregressive paths were significant and demonstrated high stability over time (B = 0.98, *p* < 0.001), which suggests there was little change in individual’s relative standing in BMIz over time. Within−time correlations revealed that dietary restraint was positively associated with concurrent weight at grade 4 and was trending toward significance at grade 3 (grade 3: B = 0.03, *p* = 0.05 grade 4: B = 0.03, *p* = 0.002). In sum, weight status was highly stable from grade 3 to grade 4, and dietary restraint was moderately stable across this timespan; weight status was concurrently associated with dietary restraint in grade 4, but not grade 3.

#### 3.1.2. Cross-Lagged Model

**Figure 2:**
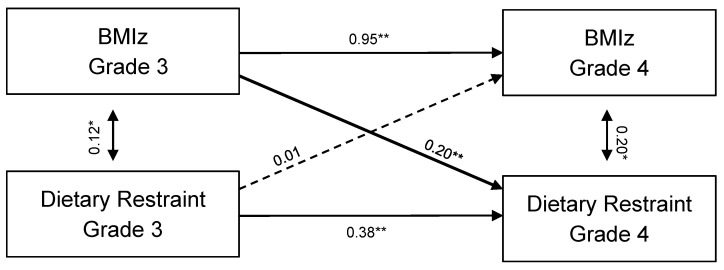
Cross-lagged model with BMIz and dietary restraint among children from grade 3 to grade 4. * *p* < 0.05. ** *p* < 0.01.

In models 2 and 3, we added cross-lagged paths from grade 3 dietary restraint to grade 4 BMIZ and grade 3 BMIz to grade 4 dietary restraint, respectively. Both models showed good model fit (see Table 4), however, cross-lagged paths from grade 3 dietary restraint to grade 4 BMIz (model 2) were non-significant. In contrast, results indicated a significant cross-lagged association from grade 3 BMIz to Grade 4 dietary restraint (model 3). Model 4 with all autoregressive and cross-lagged structural paths incorporated showed analogous results to model 3. A chi-square difference test between the stability model and model 4 was significant ((2) = 10.80, *p* < 0.01) indicating the data fit cross-lagged model better. Within the full cross-lagged model, all autoregressive paths were positive and significantly different from zero, indicating that both dietary restraint and BMIz were stable over time (Figure 2). The cross-lagged path coefficients revealed that BMIz in grade 3 was related to greater dietary restraint in grade 4 (B = 0.20, *p* = 0.001).

## 4. Discussion

In the current study, a transactional design was utilized to examine the longitudinal associations between dietary restraint and weight in a sample of elementary school-aged children. This design was advantageous in that it allowed for the examination of the direction and nature of the relation between objectively measured weight and self-reported dietary restraint across two time points during middle childhood, a developmental period that has been largely understudied in terms of the emergence of weight-control behaviors. The cross-lagged model supported our hypothesis and revealed a transactional relation between weight status and dietary restraint such that greater BMI in the 3rd grade was associated with greater dietary restraint by 4th grade.

Our findings showed that both BMI and dietary restraint scores were stable between the 3rd and 4th grade. These patterns are consistent with previous longitudinal research with this age group [31,56]. For example, Shunk and Birch (2004) explored the validity of dietary restraint as measured among girls between the ages of 5 and 9 and observed a significant correlation in scores for dietary restraint measured using the DEBQ at ages 7 and 9 [31]. It was hypothesized that girls younger than 7 did not yet possess the self-regulatory skills necessary to inhibit the cognitive impulse to obtain immediate gratification in favor of more delayed reward, making it difficult to successfully employ dietary restraint. However, by age 7, children have mostly developed the use of cognitive strategies to resist temptations allowing them the ability to practice dietary restraint [31].

As hypothesized, we demonstrated that higher weight in third grade was associated with greater dietary restraint by fourth grade. This finding remained unchanged even after controlling for child’s sex, race/ethnicity, weight in first grade, and restraint in grade 3, suggesting that child’s current weight is likely to trigger the engagement in higher levels of restrained eating and not vice versa. Our findings align with previous longitudinal research among children, which suggest that dietary restraint is a consequence of higher weight or weight status and that this association can be observed as early as elementary school-aged children [28]. Dietary restraint was once believed to emerge during adolescence and represent a consequence of the normative weight gain associated with growth and pubertal development [57,58]. Increasing weight and/or weight status among adolescents has been strongly associated with negative body image and body dissatisfaction among this population [59,60]. In turn, adolescents who are dissatisfied with their weight or appearance are more likely to engage in unhealthy weight loss behaviors, such as fasting, bingeing, and purging [38,61,62]. Similar relationships between dietary restraint and unhealthy/extreme weight control behaviors have been observed among adult populations, prompting some researchers to associate dietary restraint with unhealthy forms of dietary restriction [22,63]. However, given that the capacity for self-regulation and self-control are still developing in childhood, it is less likely that the patterns between dietary restraint and weight reflect a similar negative form of weight loss behavior among children [11,21,36]. Alternatively, the relationship between dietary restraint and weight among children could be a reflection of parental modeling and social desirability to achieve the “thin ideal” [26,64]. Studies with pre-school aged children have shown individuals in this age group have already started internalizing pressures to be thin [26]. Future research is warranted to better understand whether restrained eating in childhood could be promoted as a tool to maintain a healthy body weight through the lifespan.

In recent years, longitudinal research with adults has shown that increases in restraint over time may be associated with greater weight loss, but this relation has not been reproduced in studies among children or adolescents [11,65]. In a study involving a clinical sample of adolescents with overweight and obesity participating in a lifestyle intervention, higher baseline dietary restraint scores predicted a lower BMI z-score reduction across the 12-month study period [66]. In our study, the cross-lagged effects between dietary restraint in grade 3 and BMIz in grade 4 were non-significant, meaning dietary restraint was not associated with a change in weight (BMIz) in grade 4. There are several possible explanations for this finding. Since the restraint scale of the DEBQ is a measure of the intention to control food intake, it is possible that children in this study did not actually reduce energy intake enough to effectively regulate weight. It is also possible that individuals who report higher levels of dietary restraint are more prone to overeating, and thus are at a higher risk for weight gain. Further research is needed to examine different levels of dietary restraint and to better understand the mechanisms behind dietary restraint as a method for regulating for food intake and successful healthy weight management.

There were several strengths to the present study including the large sample of boys and girls from elementary schools, with high proportions of children with lower socio-economic backgrounds. The longitudinal design also allowed for examining changes and nature of the relation between restraint and weight over two time points during middle childhood. Furthermore, since we used a transactional model, we were able to test for stability in these measures over time and determine the cross-construct relations that emerge over and above the contributions of the stability of these constructs over time. Thus, we can conclude that our result of weight predicting dietary restraint over time was not just attributable to overweight or obese children having higher restraint scores at each time point.

Despite the many strengths of this study, it is not without limitations. First, although dietary restraint was assessed using a measure previously established in research with children of similar age, the measure still relies on self-report and thus may be subject to reporting bias. Other limitations with this tool exist regarding the ability of the DEBQ- restraint scale to adequately assess restriction and self-regulation of food intake. Furthermore, dietary restraint was not considered in conjunction with actual dietary intake, so interpretation of this measure is limited to perceived restraint and not actual caloric reductions via food intake. Secondly, our sample consisted of children from rural communities in Oklahoma, thus generalizability of the results is limited. Thirdly, the present study assessed restraint and BMI on two occasions during middle childhood, and the time points were relatively close together. Additional time points that span across middle childhood and adolescence should be used in future research because they may better capture changes in eating behaviors and associations with normative weight gain, including later changes that are associated with puberty. Finally, there are other variables that are known to influence energy balance in children, such as physical activity, which were not included in these models. Future research should examine these associations across developmental time periods to get a more accurate assessment of the relation between weight status and restraint over time.

## 5. Conclusions

The current study contributes to the growing body of literature on the development of eating behaviors in childhood and supports the role of children’s weight status in the development of dietary restraint. Understanding what contributes to higher levels of dietary restraint in childhood is important because increased dietary restraint has been linked with both successful prevention of weight gain and higher diet quality later in adulthood [16,22,65]. In our model, there was a dominant cross-lagged effect of weight status on future restraint, even after controlling for sex, race, and weight status in first grade. Rather than driving BMI trajectories upward (or downward), dietary restraint was not significantly related to future weight status in children. This has important implications for childhood obesity interventions. It is possible that certain weight-control behaviors, such as dietary restraint, can be employed in childhood to prevent excess weight gain. Future research should focus on other specific behaviors that contribute to healthy weight management in children at risk for developing obesity.

## Figures and Tables

**Table 1:** Baseline characteristics of the study cohort.

Age, Years (Range)	9.34 (8.51–10.73)
Girls	114 (43.4)
Race/ethnicity	
American Indian	48 (18.1)
African American	5 (1.9)
Hispanic	8 (3.0)
Asian	1 (4)
White	196 (74.0)
Multiethnic	7 (2.6)
Weight Status	
Normal weight	166 (61.9)
Overweight	46 (17.2)
Obese	44 (16.4)
Severe Obese	12 (4.5)
*n* = 263, expressed as *n* (%)	

**Table 2:** Descriptive information for main study variables (*n* = 263).

	M	SD	Range	Skewness
Grade 3 BMIz **^a^**	0.65	1.13	−2.2–2.7	−0.23
Grade 4 BMIz	0.66	1.23	−2.6–2.72	−0.28
Grade 3 Restraint	1.52	0.24	0.88–2.80	0.71
Grade 4 Restraint	1.45	0.22	0.89–3.00	0.96

**^a^** BMIz = Body Mass Index z score.

**Table 3:** Associations between BMIz and Dietary Restraint in Grade 3 and Grade 4.

Variable	1	2	3	4
1.Grade 3 BMIz	---			
2.Grade 4 BMIz	0.95 ***	---		
3.Grade 3 Restraint	0.26 ***	0.25 ***	---	
4.Grade 4 Restraint	0.29 ***	0.34 ***	0.43 ****	---

*** *p* < 0.001. **** *p* < 0.0001.

**Table 4:** Model Fit and Model Comparisons.

Model	χ^2^	df	CFI	RMSEA	SRMR	Comparison	Δχ^2^	Δdf
Model 1 **^a^**	15.17 *	8	0.99	0.06	0.06			
Model 2 **^b^**	14.66 *	7	0.99	0.06	0.06	M1–M2	0.41	1
Model 3 **^c^**	3.69 *	7	1	0	0.01	M1–M3	9.51 **	1
Model 4 **^d^**	3.47	6	1	0	0.01	M1–M4	10.80 **	2

**^a^** stability model. **^b^** stability model with cross-lagged paths from grade 3 dietary restraint to grade 4 BMIz. **^c^** stability model with cross-lagged paths from grade 3 BMIz to grade 4 dietary restraint. ^d^ full cross-lagged model. * *p* < 0.05. ** *p* < 0.01.

## Data Availability

The data presented in the current study are not publicly available due to the stipulations related to privacy and confidentiality in the approved IRB protocol. However, the data are available upon request from the corresponding author (LHS).
